# Effect of Water Nutrient Pollution on Long-Term Corrosion of 90:10 Copper Nickel Alloy

**DOI:** 10.3390/ma8125443

**Published:** 2015-11-27

**Authors:** Robert E. Melchers

**Affiliations:** Centre for Infrastructure Performance and Reliability, The University of Newcastle, Callaghan, New South Wales 2308, Australia; rob.melchers@newcastle.edu.au; Tel.: +61-2-4921-6044

**Keywords:** long-term corrosion, copper alloys, Cu-Ni, bi-modal, microbiologically-influenced corrosion (MIC)

## Abstract

Due to their good corrosion resistance, copper and copper alloys such as 90:10 Cu-Ni are used extensively in high-quality marine and industrial piping systems and also in marine, urban, and industrial environments. Their corrosion loss and pitting behaviour tends to follow a bi-modal trend rather than the classic power law. Field data for 90:10 copper nickel immersed in natural seawater are used to explore the effect of water pollution and in particular the availability of critical nutrients for microbiologically induced corrosion. It is shown, qualitatively, that increased dissolved inorganic nitrogen increases corrosion predominantly in the second, long-term, mode of the model. Other, less pronounced, influences are salinity and dissolved oxygen concentration.

## 1. Introduction

**Figure 1:**
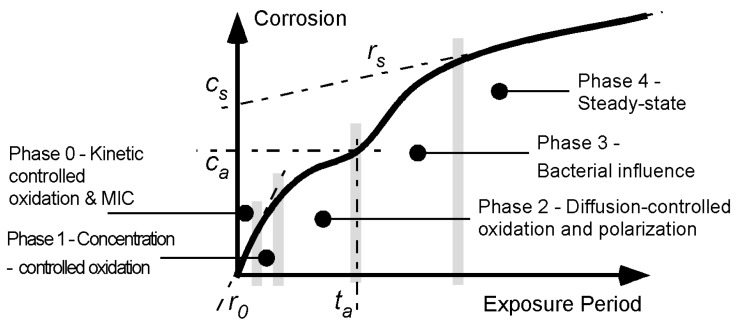
Bi-modal model for development of corrosion loss as a function of increased exposure time [2]. The first mode is from time 0 to *t_a_* and this is followed by the second mode. The parameters (*r_0_*, *t_a_*, *c_a_*, *c_s_* and *r_s_*) used to describe the model are shown.

Copper and copper alloys have good corrosion resistance and are widely used for high quality applications in the marine industry. Particularly copper-nickels (CuNi) such as 70:30 and 90:10 are much used for piping in the shipping industry when long-term durability is required. Due to the lower cost of the 90:10 CuNi alloy compared to the traditional 70:30 CuNi, it has received research attention relatively recently, to establish both its short and its longer term corrosion behaviour [1]. That work focussed mainly on phenomenological aspects and did not try to establish trends and the effect of various environmental influences. Recently, using data available in the literature, it was shown that the corrosion loss trends of various copper alloys consistently show a bi-modal corrosion characteristic (Figure 1), provided exposures over several years are considered [2]. The bi-modal functional form is considerably different from the traditional power law function commonly described in the corrosion literature (e.g., [2]). It was inherited as a simplification of the original theoretical work by Tammann [3], who was interested in the atmospheric corrosion of copper roofs. Traditionally such roofs were almost pure copper. As it happens, for pure copper exposed to pure water (e.g., as approximated by rainwater in unpolluted environments) the bi-modal function degenerates into a simpler mono-modal function that can be approximated by the power law [2]. More generally, however, the recent work [2] showed that the power law usually is not applicable for copper alloys.

In showing that the bi-modal model also applies to copper alloys, the earlier study [2] used data from a variety of sources, including that from a five year exposure study of 90:10 copper-nickel at 14 sites world-wide [1]. Attention was focused only on whether the bi-modal form was evident in the data for the various copper alloys. No attention was given to the relative magnitudes of the first and the second mode, or to the values of the parameters shown in Figure 1 and how these might be related to environmental and possibly material factors. For steels, it has been shown that water quality, and in particular the concentration of nutrients critical for microbiological activity, has an important effect on the long-term corrosion of steels in immersed and tidal zone exposure conditions [4]. Copper nickels are known to be prone to microbiologically-influenced corrosion (MIC) and, thus, a parallel long-term effect might be expected. The next section gives a brief background summary of the corrosion of copper alloys. Data for the corrosion of a 90:10 CuNi alloy (C70600) is then considered relative to the (limited) information available for the exposure conditions at the corrosion test sites of the world-wide study [1]. This includes the conditions likely to have been operational at each site at the time the test program was carried out. Comparative comments are then made about the corrosion behaviour of 90:10 CuNi relative to environmental influences. The main focus is on the effect for long-term corrosion.

## 2. Background

For steels, the trend in corrosion mass loss most consistent with data for exposures extending over many years is a bi-modal functional relationship [4]. It is summarized in Figure 1 together with the variables that parameterize the main parts in the model. On the basis that there are known similarities in the corrosion of metals, a considerable body of data was used to show [5] that the corrosion loss (and pit depth) of aluminium alloys exposed to a variety of wet corrosion conditions also have bi-modal corrosion (and pit depth) behaviour. As noted, recently this conclusion was extended to the corrosion behaviour of copper alloys [2].

The bi-modal model consists of a number of sequential phases, each describing a (idealized, dominating) corrosion process. Initially (*i.e.*, in Phase 0, Figure 1) surface reaction kinetics governs the rate of corrosion. These may be influenced by some degree of biofilm, algal, or microbiological colonization. After only hours or days this is followed by the corrosion rate being limited by the rate at which the oxygen necessary for the corrosion process, can diffuse out of the local water. This is Phase 1. There are various descriptions (such as the Nernst barrier, boundary concentration control mechanism, concentration polarization) [6]. After some days (weeks in cold waters) this mechanism is superseded by Phase 2 in which the corrosion rate is limited by the diffusion of oxygen through the corrosion products that build up on the external surface as a result of corrosion. The diffusion process becomes increasingly more difficult as the rust layers become thicker and less permeable. During the whole of this period of exposure the corrosion process involves the formation of corrosion pits. These are known to be a significant component of early corrosion loss [7].

As shown in Figure 1 the three early Phases 0–2 of the corrosion processes tend to follow a relatively smooth curve for corrosion loss as measured by mass loss. The overall trend usually can be fitted reasonably well by a simple monotonic function such as the power law *c*(*t*) = A*t*^B^ where *c*(*t*) is the corrosion as a function of time *t* and A and B are constants obtained empirically by fitting the function to a set of data. However, it is known that the “constants” A and B are sensitive to the time span of the data, that is, the length of time over which the power law is fitted [8].

**Figure 2:**
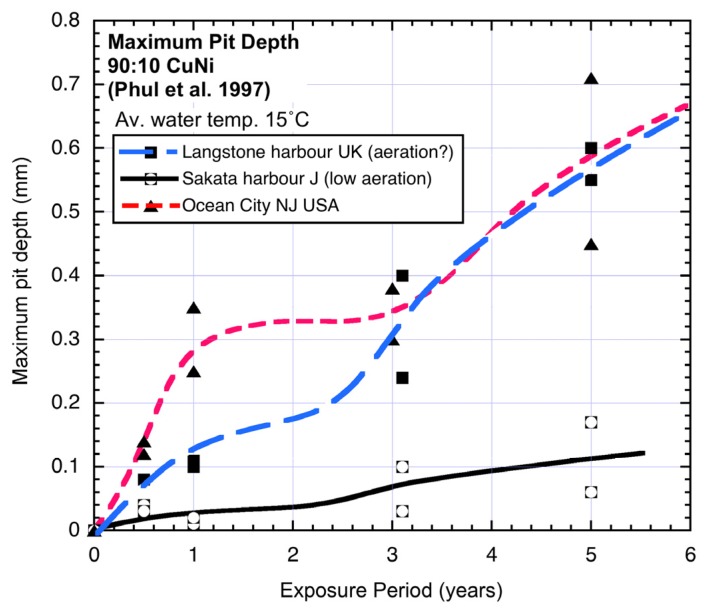
Typical bi-modal trends for pit depth. Data, but not trends and interpretation, from Phull *et al*. 1997 [1]. As shown, for Langstone harbour there is some question about the degree of aeration of the seawater.

For many data sets obtained from relatively short term corrosion experiments the power law can be considered a sufficient model. In these cases the exposure period is not long enough to start to set up the conditions necessary for the development of the second mode in Figure 1. The second mode applies after *t_a_* (Figure 1). It is idealized initially by Phase 3 and in the long term by Phase 4. Based on the corrosion behaviour for steels, both involve predominantly low oxygen corrosion conditions [4]. Figure 2 shows three examples of the bi-modal functional form for the pit depth of 90:10 copper nickel alloys [2]. 

For steels and for aluminium the second mode of the bi-modal characteristic involves hydrogen evolution from corrosion pits under rust products and this is possible because hydrogen can displace the metal ion, as can be determined from free energy considerations [9] or from the relevant Pourbaix diagram [10]. A similar situation is likely to exist for copper nickel alloys, as these obviously are not pure copper.

A theoretically important exception is the corrosion of pure copper in pure water, for which hydrogen cannot, according to free energy considerations, displace the copper ions, although there are some experimental observations that claim there could be a very slow reaction [11]. However, others claim not to have observed such a reaction [12]. The subject is controversial. Herein, attention is confined to copper alloys and in particular to 90:10 copper nickel (CuNi).

An important aspect of the corrosion of copper alloys is that they are prone to microbiologically-influenced corrosion (MIC) particularly in seawater exposures. Copper alloys have been known for many years and from many experimental and practical observations to show much more severe corrosion when the seawater is polluted with sulphides [10,13,14,15,16]. Mostly the investigations have been confined to inorganic sources of sulphides, but increasingly it is being recognized that sulphides also may be generated as a result of the metabolism of sulphate reducing bacteria and potentially other prokaryotes [16]. Although copper is known to be toxic for macro-fouling, algae and microorganisms have been observed to colonize on copper surfaces [17] and, thus, MIC is feasible.

The conditions under which MIC of copper alloys in seawater exposures can or will occur are not clear. Unfortunately, studies reporting the longer-term corrosion of copper alloys only seldom provide detailed water quality statements and if they do dissolved inorganic nitrogen (DIN) (or its usual major components nitrate and ammonia) usually are missing [18,19,20]. Presumably this is because nitrates and ammonia had not been associated with the corrosion products created by the usual electro-chemical reactions. Another factor is that, until recently, it was considered that bacterial counts and examination of the microorganisms present in corrosion products or near the corrosion interface held the key to identification of the possibility and the importance of MIC. However, increasingly it has become clear that these approaches are not necessarily practical ways forward [21]. Until recently this was the also case for steels in seawater.

Recent developments for the effect of nutrients on the corrosion of steel are of direct relevance, however. Some success has been achieved with a classical dose-response approach. In particular, good correlation has been shown recently between elevated corrosion loss or pitting and the concentration of DIN in the local seawater [22]. This is consistent with DIN being a critical nutrient for microbiological metabolic activity in seawater and that, in turn, governs the microbiological corrosion rate. The other critical (micro-) nutrient for microbiological activity, ferrous iron [18] is readily available as a direct result of the corrosion process for steels. Other nutrients such as organic carbon and sulphates invariably are available in seawater [18,19]. It is assumed also that sufficient energy (*i.e.*, electron transfer) is already available to the microorganisms from the abiotic corrosion reactions.

It is likely that a generally similar situation is applicable for the MIC of copper alloys, noting that their composition typically includes a trace of ferrous iron [23]. However, since it is required only as a micro-nutrient, this is likely to be sufficient. For seawater MIC of copper alloys, therefore, the availability of DIN would appear to be the critical aspect.

Normally, the lack of information about water quality renders deductions about the possible influence of MIC difficult, if not impossible. As shown for steels [22] a quantitative analysis requires measured information about corrosion loss, water temperature and about water quality. For 90:10 CuNi such detailed information, particularly about DIN concentrations, is not available. This includes the data source for the present study [1]. However, as shown below, a qualitative approach can throw some light on what would be expected. It uses measured corrosion mass loss observations and informed but qualitative data for environmental conditions. To proceed, Table 1 summarizes the known (qualitative) effects of various parameters on the corrosion of copper nickel alloys [24], separated here into the effect for each of the two modes of Figure 1.

**Table 1:** Effect of main influences on CuNi corrosion mass loss. DIN: Dissolved inorganic nitrogen

Increase in Water Quality Property:	Mode 1	Mode 2
Water temperature	Increase	Increase
Salinity	Increase	Flow-on effect from mode 1
Dissolved oxygen (DO)	Increase	Flow-on effect from mode 1
Water velocity	Increase	Flow-on effect from mode 1
DIN (critical nutrient)	Usually a small increase	Large increase

## 3. Experimental Data and Trends

The data used in the present analysis is drawn entirely from the five-year ASTM study [1] of the marine immersion corrosion of 90:10 Cu–Ni (C70600) coupons (composition wt % 9.44 Ni, 1.40 Fe, 0.29 Mn, 0.008 P, 0.007 S, bal Cu). Each coupon, 300 mm × 100 mm × 6 mm in size, was oriented vertically and electrically isolated. The coupons were exposed at 14 different sites (Table 2) and recovered, two coupons at a time, nominally at 0.5, one, three, and five years of exposure. The sites and the recorded environmental conditions and water quality parameters are shown in Table 2. On recovery the samples were cleaned to conventional ASTM standards and procedures. Mass loss was then determined from the final and original masses and one-sided corrosion losses calculated. Full details are available [1]. Although the mass and corrosion losses are tabulated, they were not plotted and the possible trends in the corrosion losses were not discussed. Comments about the effects of possible influencing factors are inconclusive [1]. No information was published at the time about water quality at the 14 sites. Subsequent investigations produced the results summarized as shown in Table 3. Some of the sites were visited by the author and the comments are based in part on discussions with local experts and others. In this Table, one of the sites (JA, Sakata harbour, Japan) has been ignored because the corrosion loss results for year five are both much lower than those for the earlier exposures. No explanation for these anomalous results was given.

**Figure 3:**
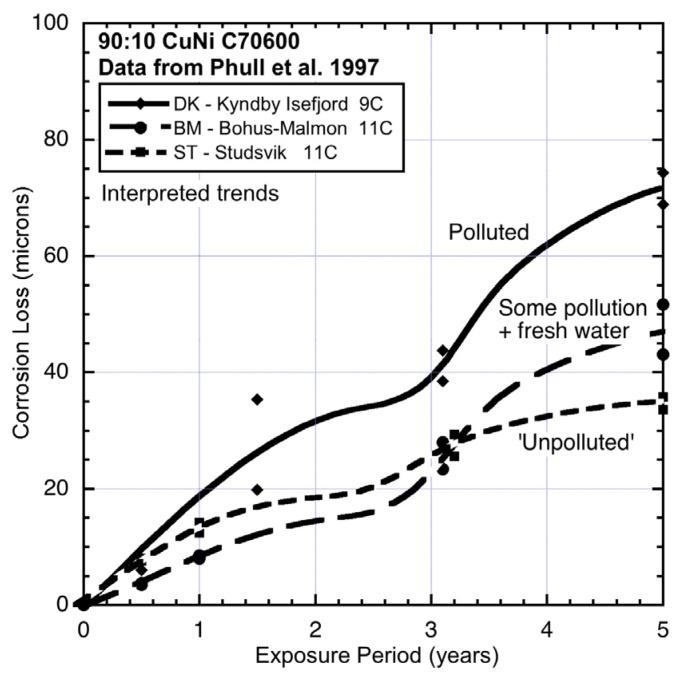
Data and interpreted trends for colder seawater sites DK, BM and ST.

**Figure 4:**
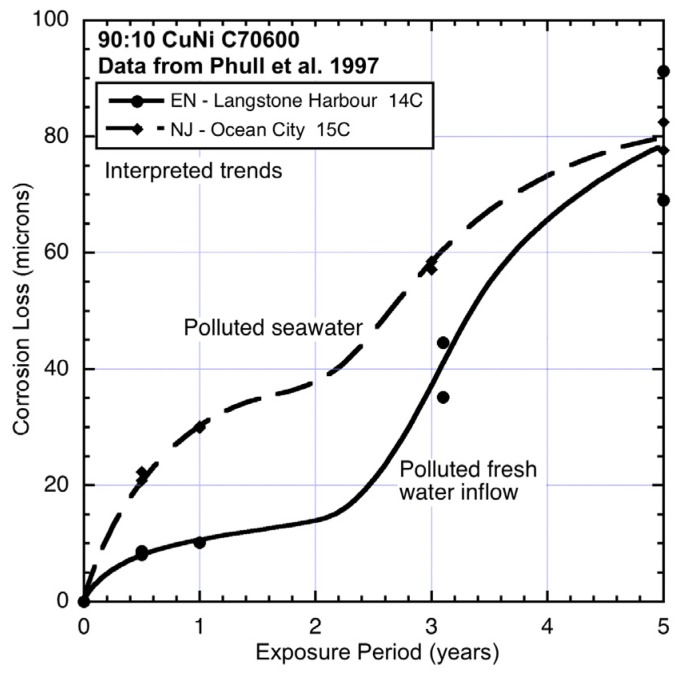
Data and interpreted trends for cool seawater sites EN and NJ.

**Figure 5:**
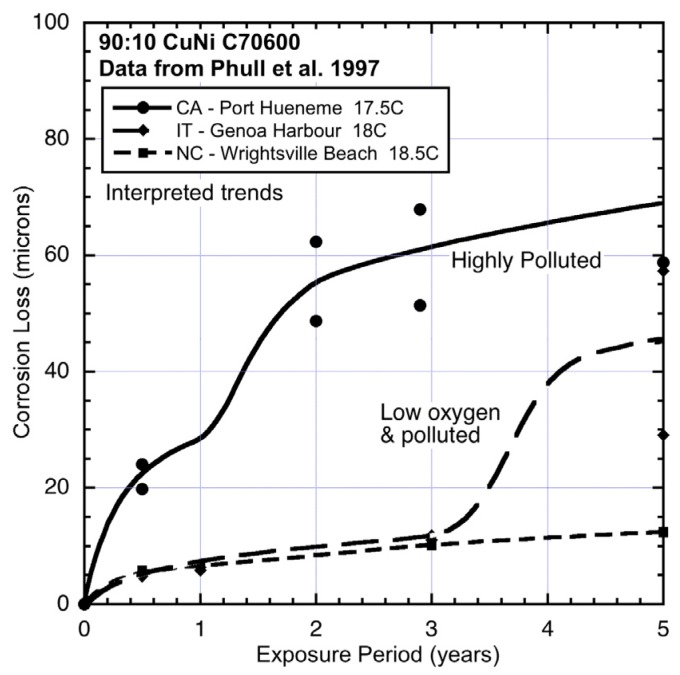
Data and interpreted trends for temperate seawater temperature sites CA, IT and NC.

**Figure 6:**
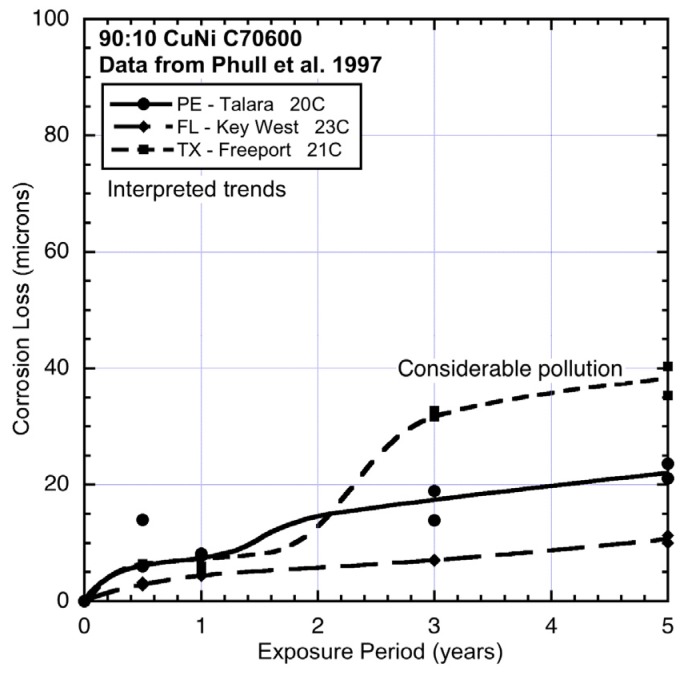
Data and interpreted trends for temperate seawater temperature sites PE, FL and TX.

**Figure 7:**
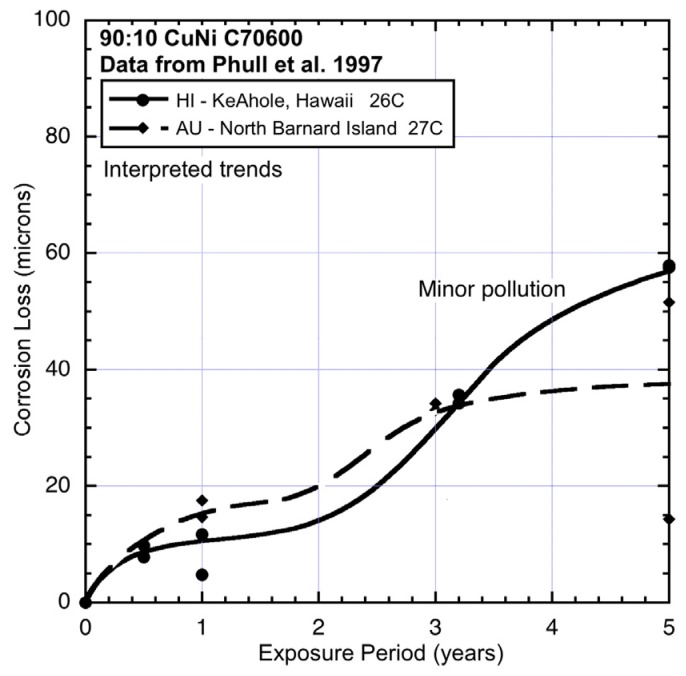
Data and interpreted trends for warm seawater sites HI and AU.

The corrosion loss data for 90:10 CuN extracted from the ASTM study [1] and the trends interpreted from the data are shown in Figure 3, Figure 4, Figure 5, Figure 6 and Figure 7, ordered by average seawater temperature. In each case trends based on subjective interpretation of the data are shown, based on the assumption that the data set in each case is bi-modal. 

**Table 2:** Exposure sites and basic seawater data ordered by mean seawater temperature [11].

Key	Geographic Location	Temperature (°C)	Mean Temperature (°C)	DO (ppm)	Salinity (mg/L)	pH
DK	Isefjord, Denmark	0–18	9	NA (low)	18–28	7.5–8.0
BM	Bohus-Malmon, Sweden	2–20	11	6–10	21–28	8.0–8.2
ST	Studsvik, Sweden	2–20	11	6–10	7.8–8.1	7.4–7.6
EN	Langstone Harbour, UK	5–22	14	8.8–11.8	34–34.6	8.0–8.2
NJ	Ocean City, NJ, USA	1–29	15	5.2–11.7	31–34	7.5–8.2
CA	Port Hueneme Harbour, CA, USA	14–21	17.5	3.6–5.3	33	7.9–8.1
IT	Genoa Harbour, Italy	11–25	18	5.8–8.9	38.2–36.6	8.1–8.3
NC	Wrightsville Beach, NC, USA	7–30	18.5	5.0–9.6	31.8–37.6	7.9–8.2
PE	Talara, Peru	18–22	20	5–6	35.8	8.2
TX	Freeport, TX, USA	15–27	21	1.5–6.0	21.1–35.3	7.5–8.6
FL	Key West, FL, USA	16–31	23	4–8	33–39	8.0–8.2
HI	Keahole, HI, , USA	24–28	26	6–14	34.6–35	8.0–8.3
AU	N. Barnard Is, Australia	21–30	27	5.1–6.5	31.7–37.2	8.2–8.3

**Table 3:** Qualitative descriptions of water quality for exposure sites [11].

Key	Descriptive Water Quality
DK	Enclosed seawater site with some fresh water inflow likely.
BM	Coastal site, some pollution from adjacent harbour likely.
ST	Coastal site, likely minimal local pollution likely.
EN *	Enclosed bay, some pollution and freshwater inflows likely.
NJ *	Heavily polluted with sewage effluents.
CA	Heavily polluted with sewage effluents and military wastes.
IT	Likely heavily polluted.
NC *	Banks Channel seawater, some freshwater and some sewage effluent.
PE	Coastal harbour site, pollution likely very low.
TX	Channel within industrial plant.
FL *	Open site, very low pollution levels.
HI *	Deep open water coastal site but coupons located on effluent pipe.
AU *	Open ocean site, very low coastal pollution.

Note: * personal field inspection.

## 4. Analysis

The data sets in Figure 3, Figure 4, Figure 5, Figure 6 and Figure 7 have been grouped by average seawater temperature. In each group (*i.e.*, in each Figure) it is seen that there are considerable differences between the trends. Nevertheless, it is evident that apart from some with very low levels of corrosion for which the exact trend form cannot easily be discerned, all the trends show bi-modal behaviour for corrosion loss as a function of exposure period. While other trends might be constructed through the data, the bi-modal functional form is always a plausible choice and is, in any case, consistent with the observations earlier for copper alloys more generally [2].

As shown in Table 1, apart from seawater temperature there are several other possible influences that can affect corrosion loss. This means that, in principle, comparisons should be based on some type of vectorial analysis process. However, there is clearly insufficient data available for such a formal approach. To make progress, an alternative approach is to consider the individual influences separately and only later consider possible interactions. It is convenient to start with sites for which the seawater is unlikely to have been diluted with fresh water, pollution is likely to have been low, and water velocity to have been moderate to low. The latter means that dissolved oxygen (DO) is likely to be typical for seawater at a given temperature. The sites most likely to fall in this category are FL (Florida) (Figure 6) and AU (Australia) (Figure 7), both well away from sources of pollution.

Comparing the data and trends in Figure 6 shows that the corrosion losses for FL are considerably lower than those at the two other sites, even though these have very similar average seawater temperatures. Site PE (Peru) (Figure 6) is a coastal harbour site and thus likely to have had some degree of pollution but, because the site is relatively open, the other environmental conditions are likely to have been similar to FL. This is reflected in the modest increase in corrosion loss in mode 1 and also in mode 2, most likely the result of modest nutrient pollution at the site. In contrast, the corrosion losses and the trend for site TX (Texas) show much higher corrosion loss in mode 2. This is consistent with the known high level of pollution at this site [1,25].

Comparison between AU and HI (Hawaii) (Figure 7) shows that corrosion loss in mode 1 at HI was lower, which suggests lower salinity. This possibility is consistent with the close-to-shore location of this site and the fact that the coupons were mounted on top of an outfall pipe [1]. This also is the likely explanation for the higher corrosion loss in mode 2 for HI, with nutrient pollution, likely because of the effluent from the outfall. As for TX, the main effect here is increased corrosion loss in mode 2.

**Figure 8:**
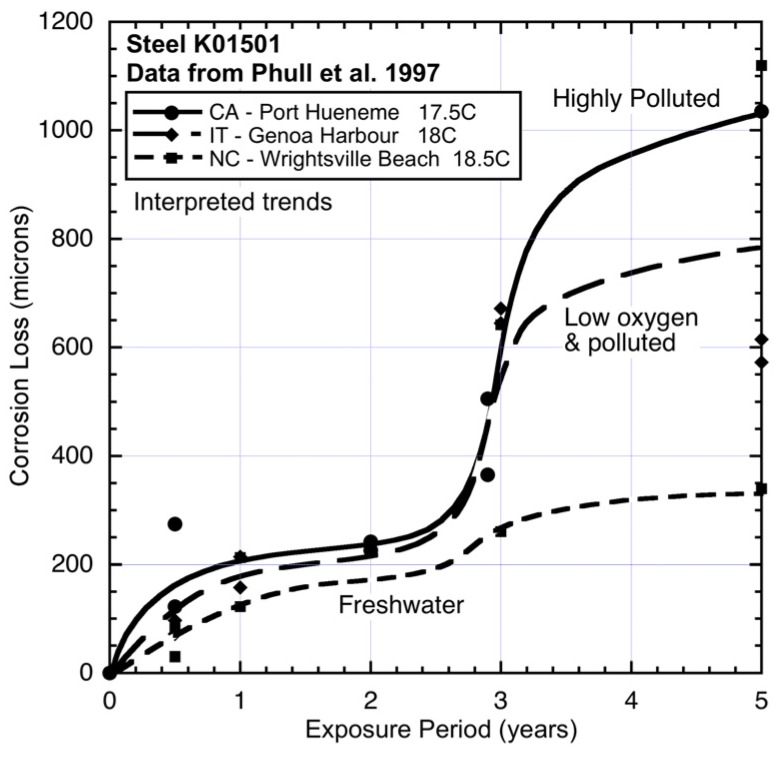
Corrosion loss data and trends for low alloy steel (type K01501) at CA, IT and NC showing effect of increasing pollution (nutrient) concentration in the seawaters. The freshwater contribution to the seawater at NC lowers the early corrosion losses. Data from [1].

Compared to FL (Figure 6), the corrosion loss at NC (North Carolina) (Figure 5) is somewhat greater even though the average water temperature is a few degrees (4.5 °C) lower (Table 2). In this case, too, it is known [1,25] that the seawater at this site (Wrightsville Beach, NC, USA) is periodically polluted by sewage overflows. However, there is also freshwater inflow, and the results suggest that these tend to cancel. The other two sites shown in Figure 5 have similar water temperatures but show very different corrosion loss trends. Site IT (Italy), Genoa Harbour, is almost certainly highly-polluted and very likely to have low salinity. Quantitative data for these factors could not be obtained for the period of interest. Since nutrient pollution has some effect throughout the corrosion processes, the low level of corrosion in mode 1 most likely indicates reduced oxygen availability and/or reduced salinity, even though this is not evident from the information as reported (Table 2). The very large increase in corrosion loss after about three years’ exposure, *i.e.*, in mode 2, is consistent with high pollution levels. Generally similar corrosion loss behaviour occurs also for steel exposed at this site (Figure 8).

For site CA (California), which is adjacent to the US naval facility at Port Hueneme, CA, there is much anecdotal information (but little available quantitative data) that the site was highly polluted at various times and presumably also during the study period [1,25]. Corrosion of steels at this site also has been noted as very high [25] and this is seen also copper bearing steel (Figure 8) exposed in parallel to the CuNi test program. Figure 8 also shows, for comparison, the data and trend for corrosion of steel at NC (Wrightsville Beach). Similar to the effect on 90:10 CuNi, despite some freshwater inflow to the corrosion test site, and which would result in the seawater being somewhat brackish, the corrosion loss trend can be considered reasonably close to that for an unpolluted site. The freshwater contribution tends to lower the early (*i.e.*, mode 1) corrosion losses and since the level of water pollution is relatively small [25], there is little influence on the corrosion loss in mode 2, both for steel (Figure 8) and for 90:10 CuNi (Figure 5).

At the two cool water sites EN (England) and NJ (New jersey) (Figure 4), the corrosion loss trends are very different. The EN site is in an almost enclosed bay with low water velocities and little wave action and receiving nominally fresh water from small local watercourses and storm-water drains. Periodic periods of lower salinity are, therefore, likely (*cf.* Table 2). This may explain the relatively low corrosion in mode 1. However, the high corrosion losses in mode 2 (Figure 4) suggest high levels of water pollution and, given the surrounding areas are mainly domestic dwellings and little industry, likely to be, mainly, nutrient pollution. On the other hand, site NJ, which is off Ocean City, is known to be quite polluted [25] and this appears to be reflected in the high corrosion losses both in mode 1 and in mode 2. The site is exposed and thus subject to wave and current action, as well relatively high dissolved oxygen levels (*cf.* Table 2).

**Figure 9:**
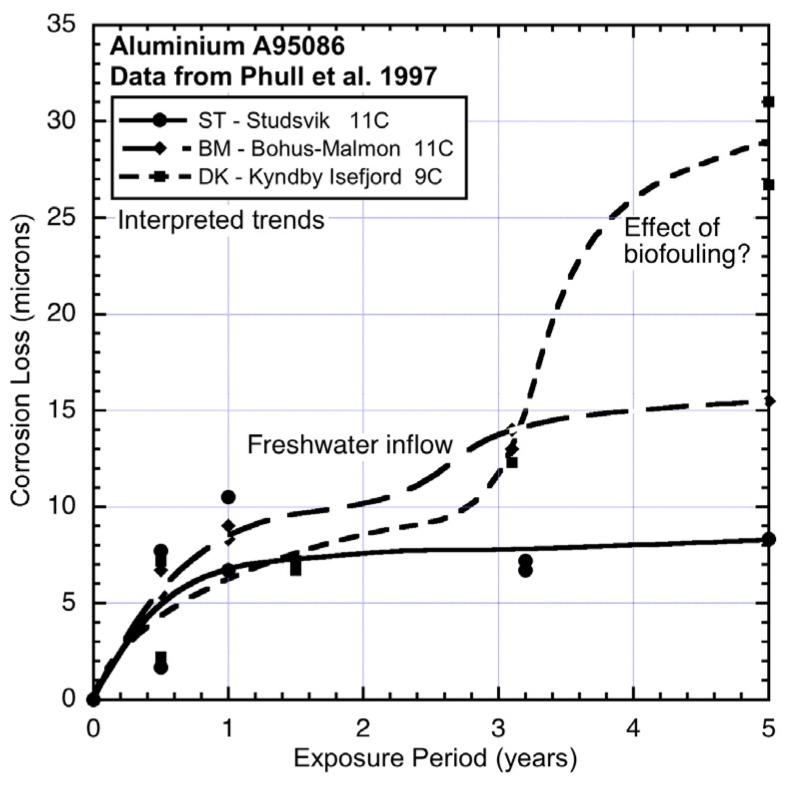
Corrosion loss data and trends for aluminium (type A95086) at DK, BM and ST showing effects of salinity and of gross biofouling. Data from [1].

Finally, the three sites with the coldest waters and for which the corrosion data and trends are shown in Figure 3, all show quite high corrosion losses in both modes 1 and 2. Sites BM and ST show similar corrosion losses for mode 1, although the slightly lower losses for BM are likely to be the result of some lower level of salinity at this location. This is supported by the corrosion loss trends for aluminium exposed at this site (Figure 9), noting that aluminium corrosion increases with lower salinity [24]. Figure 9 also shows that for site DK mode 2 for aluminium can be quite high. The reason for this high corrosion loss remains to be determined but it can be speculated that it is attributable to severe localized corrosion that is likely under deposits or growths in close contact with the metal, such as biofouling that is not removed from the surfaces of metals. Aluminium is known to be prone to be subject to intense corrosion attack under deposits [23].

As evident from Figure 8 and Figure 9, the ASTM study from which the data for 90:10 CuNi used herein was drawn also considered steel and aluminium. The results for the steel have been used in earlier analyses [22,25] and need not be discussed here. Some of the data for aluminium also have been used earlier without specific attention to MIC since this is unlikely to be significant in seawater [5]. A detailed study of the data for all the 14 sites, while desirable, is hampered by the lack of detailed environmental data for the exposure sites. This obviously is a problem also with analysing the CuNi data, except that both steel and CuNi are prone to MIC and have a similar response to salinity. For aluminium there appears to be no known effect of MIC in seawater and a different response to salinity and, as noted, it is much more prone to localized attack under deposits (or other) that create local oxygen-deficient areas.

## 5. Discussion

**Figure 10:**
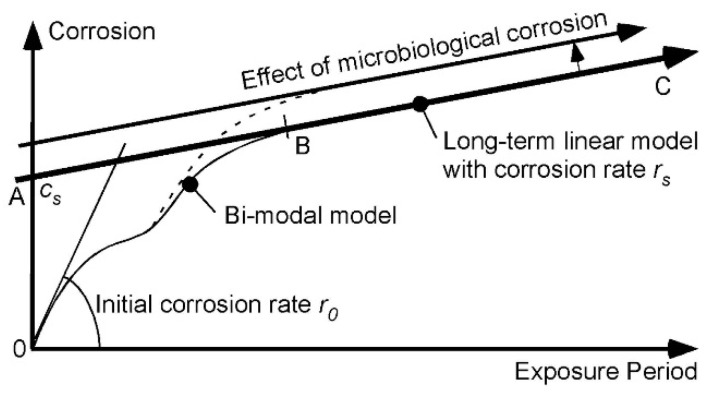
Simplified (linear positive definite) corrosion loss model for long-term corrosion of 90:10 CuNi, showing main effect of nutrient pollution and consequent microbiological corrosion.

Overall, Figure 3, Figure 4, Figure 5, Figure 6 and Figure 7 demonstrate that despite there being available only four data points for each trend, the trend in most cases is clearly bi-modal and in most other cases can be constructed as bi-modal. This is consistent with the earlier findings for 90:10 CuNi that it follows the same trend as earlier shown to hold for other copper alloys [2]. As can be seen from the trends in Figure 3, Figure 4, Figure 5, Figure 6 and Figure 7, and from consideration of water (and hence likely nutrient) pollution, the primary time when MIC is most severe for CuNi is in Phase 3 (Figure 1). This means that the bi-modal model for long-term corrosion loss can be simplified to the linearized model shown in Figure 10, also proposed for the simplified consideration of MIC for steels [22]. It is applicable, strictly, only after point B and, thus, only for long-term corrosion. For shorter-term corrosion the bi-modal part before point B must be used. The definition of B will depend, of course, on the calibration of the bi-modal model to actual data and the influences on corrosion—This is a matter for further research.

As noted earlier [9], any MIC effect is additional to already active abiotic electrochemical corrosion. This is because, as noted, the possibility of bacterial contribution to corrosion requires existence of a set of appropriate conditions and this includes the availability of nutrients necessary for microbial metabolism. In seawater, these usually are readily available, apart from DIN and ferrous ions [19]. As noted also, most coppers and copper alloys have trace amounts of Fe and, thus, this is unlikely to be a limiting (micro-) nutrient.

Comparison of the corrosion loss trends between the sites at similar seawater temperatures and the information in Table 2 and Table 3 about the conditions at each site supports the inference that for some sites there is evidence of the influence of MIC fostered by water pollution, and by implication by DIN. The fact that microbiological influences can be important for the corrosion of copper nickels is already well-known. However, the present work shows that the major effect is for Phase 3 (Figure 1) with consequent influence on Phase 4, and that in severe cases there also is a strong influence earlier (*i.e.*, in mode 1). This is demonstrated in the trends in Figure 3, Figure 4, Figure 5, Figure 6 and Figure 7.

For steels, the effect of salinity, DO, and MIC have been explored using the available data sets [20,22]. MIC in particular has been related to the availability (concentration) of DIN in seawater and correlated with immersion corrosion of steels. Unfortunately, the quantitative information about the corrosion of 90:10 CuNi, and particularly the environmental parameters such as salinity, DO and nutrient concentrations and how these vary throughout the year is very limited. It is insufficient to develop correlations between corrosion and the environmental parameters. To do so properly requires quantitative data for environmental and microbiological parameters at exposure sites, similar or better than has been used previously for steels [22]. The provision of such much more detailed information for CuNi is an open challenge. The same applies for the possible effect of galvanic coupling between Cu and Ni and microstructural effects, as already well-known for short-term corrosion [10].

## 6. Conclusions

The development of corrosion loss with time for copper alloys and in particular for 90:10 CuNi is bi-modal as a function of time of exposure. As for steels, that function can be simplified, for long-term corrosion loss, to a linear function that is positive definite at time zero. The present results also demonstrate qualitative correlation between increased water pollution and increased corrosion loss. The corrosion loss in the early part of the second mode (*i.e.*, Phase 3) of the bi-modal model is most sensitive to the effect of water pollution and this is carried on in the longer term. This effect can be interpreted to be the result, largely, of microbial activity. Quantitative calibration of the model and the effects of environmental influences, including those of elevated seawater nutrients, remains an open question that demands high quality environmental as well as corrosion data.
